# Radiological features of central giant cell granuloma: comparative study of 7 cases and literature review

**DOI:** 10.1259/dmfr.20200429

**Published:** 2021-04-21

**Authors:** Samip Shrestha, Jia Zhang, Jun Yan, Xiaomin Zeng, Xiaoyong Peng, Bo He

**Affiliations:** 1 Department Of Medical Imaging, First Affiliated Hospital of Kunming Medical University, Kunming, Yunnan Province, China; 2 Department of CT, Xuzhou Central Hospital Jiangsu Province, Xuzhou, China

**Keywords:** Central giant cell granuloma, CGCG, giant cell granuloma, reparative granuloma

## Abstract

**Objective:**

To review and analyze the clinical and imaging features of central giant cell granuloma patients and to review the relevant literatures for the diagnosis and clinical manifestation of central giant cell granuloma.

**Methods:**

Seven cases of central giant cell granuloma were retrospectively selected for the study, all of which were confirmed by pathology and had relevant imaging investigations. All seven cases had undergone CT scan, three cases had undergone MRI scan. Detailed clinical features were compared along with the imaging findings and analysis was done on the basis of their presentation and imaging features.

**Results:**

The clinical features, radiologic features were varied according to the site of the lesion. CT features include unevenly dense expansile mass causing bone destruction and cortical thinning. While MRI features with low to iso-intensity in *T*_1_- and *T*_2_ weighted images. There may be presence of cystic degeneration, hemorrhage or hemosiderin deposits or osteoid formation, which can cause T1 and T2 signal changes. On contrast study, the lesion doesn’t enhance but periphery may enhance mildly.

**Conclusion:**

Unevenly dense expansile mass with bone destruction and cortical thinning with low to iso-intensity in *T*_1_ weighted and *T*_2_ weighted images and mildly enhance peripherally, Central giant cell granuloma should be considered.

## Introduction

Central giant cell granuloma (CGCG) is rare and locally invasive intraosseous, non-neoplastic lesion. It consists of cellular fibrous tissue that contains multiple foci of hemorrhage, aggregations of multinucleated giant cells and occasionally trabeculae of woven bone.^1^ It was first described by Jaffe in 1953 as an idiopathic non-neoplastic proliferative lesion.^2^ In the past, this condition was used to be called giant cell reparative granuloma, as it was primarily considered as a local reparative reaction of bone, possibly due to intramedullary hemorrhage or trauma. Nowadays, the term reparative has been subsequently been discontinued since the lesion are locally invasive and destructive in nature.^1,3,4^

Although the etiology and pathogenesis of CGCG is still unknown, it has been believed to be associated with local trauma, repair processes, inflammatory lesion or any development disorders.^4,5^ Giant cell granuloma is a rare bony lesion in the head and neck region. The mandibular bone is affected in 70% of cases.^4^ The imaging manifestations in clinical work are often confused with giant cell tumor of bone, aneurysmal bone cyst and brown tumor. In this study, the imaging findings of seven cases of CGCG in different parts are summarized, the aim of which is to improve the understanding of CGCG, to detect and diagnose early, and to choose reasonable treatment methods.

## Methods and Materials

A total of 19 cases of CGCG were confirmed giant cell granuloma (GCG) by pathology in our (Kunming Medical University) hospital from 2007 to 2019. The institutional review board of our hospital (First Affiliated Hospital of Kunming Medical University, Kunming Medical University, Kunming, Yunnan, China) approved this retrospective study and written informed consent was waived. All of 19 cases were reviewed retrospectively on the basis of their history, clinical features and radiologic descriptions available at our institution and only seven cases were selected for the study, as they had all the relevant imaging and pathological examination findings which included at least one of the radiological investigations with routine Non-enhanced CT scans (NECT), contrast enhanced CT scans (CECT), MRI scans and histopathological investigations.

Of these selected seven cases, histories were reviewed in each cases and general data regarding age, gender, presenting symptoms, symptom duration, past history of trauma, lesion size, appearance, radiological findings, laboratory data, treatment modalities and follow-ups were tabulated.

Radiological investigations were reviewed in each case which included CT scan, and MRI with and without contrast, histopathology investigations with hematoxylin and eosin stain. All the patients had undergone CT scan investigation and three patients had MRI scans. CT scan images were obtained by using Siemens Somatom Definition AS 128 sliced CT scanner and non-ionic iodine Iohexol was used as contrast agent for CECT. MRI scans were obtained from GE 3.0 T or Philips 1.5 and 3.0 T MRI scanners. Three patients had undergone MRI scans and image sequences obtained were axial *T*_2_ weighted image (*T*_2_WI), *T*_1_ weighted image (*T*_1_WI), *T*_2_ fluid attenuated inversion recovery image (FLAIR), sagittal *T*_1_WI, diffusion-weighted image (DWI) (*b* = 1000 s/mm2). Gadolinium-diethylenetriamine pentaacetic acid (Gd-DTPA) was used for contrast enhancement.

## Results

The mean age at diagnosis of CGCG was 22 (standard deviation 15.63) among the seven cases with age ranging from 6 to 46 years. Three cases had GCG lesion in mandible (43%), two cases had lesion in sellar (29%) and one case had lesion in temporal bone (14%) and one case had in maxillary bone (14%). The history of significant trauma to the affected region could not be found in most of the cases (six cases) and only one case had history of trauma to the head could be found.

The most common presenting symptoms were swelling and pain. Other symptoms were headache, nausea, vomiting noted in two patients with lesion in sellar region, visual disturbance and protrusion of eye as well as nasal obstruction in one patient with lesion in maxillary sinus. Two patients with mandibular involvement had complains of difficulty in chewing and swallowing.

Every case was confirmed central GCG by histopathology. The typical GCG showed clusters of benign giant cells with spindle cell proliferation between the clusters. The cells were arranged loosely without any cell atypia. There was presence of evidences of new bone formation as a result of reactive process. The new bone formed mostly depends on the osteoblastic activity.

Every case had undergone at least one of the radiological investigations among CT scan or MRI with or without contrast. All cases showed lesions significant damage to the involved bone. All the cases had well-defined margin and multilocular. The detailed information on each case are summarized in the following Table 1.

**Table 1.** Summary of data from the patients with CGCG

Case no	Imaging	Lesion site	Clinical symptoms	Lesion size	Imaging features
1	CT	Left mandible bone	h/o trauma 3 months back. Pain, swelling on left mandibular region and difficulty in eating.	5 cm x 5 cm x 3 cm	Expansile lesion with bony destruction in left mandible. Well circumscribed, radiolucent with cortical bone thinning. Slightly enhanced periphery. Adjacent soft tissue compression.
2	CT, MRI	Sellar region	Headache with nausea and vomiting	3 cm x 2.8 cm x 2 cm	Expansile uneven density mass in left sided sellar region. Peripheral bone destruction. mild uneven enhancement. Low to iso intensity in *T*_1_WI, *T*_2_WI, FLAIR images.
3	CT, MRI	Left maxilla bone	Left eyeball protrusion, blurring in vision, itching, nasal obstruction	4.8 cm x 5.3 x 5.9cm	Uneven density shadow with multiple cystic lesions with multiple fluid–fluid signals within the lesion. Bone destruction present. Left eyeball pushed outward. Uneven mild enhancement in periphery and cystic walls
4	CT	Left mandible bone	Jaw pain, swelling and difficulty in eating	2.2 x 2.1cm x 1.8 cm	Radiolucent lesion in ramus of left mandible with bone destruction. Mild enhanced periphery in contrast study. Left masseter muscle swollen.
5	CT	Right temporal bone	Severe headache, nausea, vomiting	1.8 cm x 2.1 cm	Slightly annular high-density lesion in temporal bone with mild compression of adjacent brain resulting patchy edema in the brain surrounding the lesion. Skull eroded but not perforated.
6	CT, MRI	Sellar region	Headache	1.6 cm x 2.3 cm x 1.1 cm	Expansile lesion in the Sella with bone destruction, slightly higher T2 signals and iso T1 signals. Pituitary stalk compression. Uniform enhancement present.
7	CT	Left mandible bone	Left mandibular swelling and pain and difficulty in eating	2.4 cm x 2.5 cm	Low density mass arising from anterior mandibular surface. Bone destruction and cortical thinning. No enhancement.

FLAIR, fluid attenuated inversion recovery; *T*_1_WI, *T*_1_ weighted image; *T*_2_WI, *T*_2_ weighted image.

**Figure 1.**
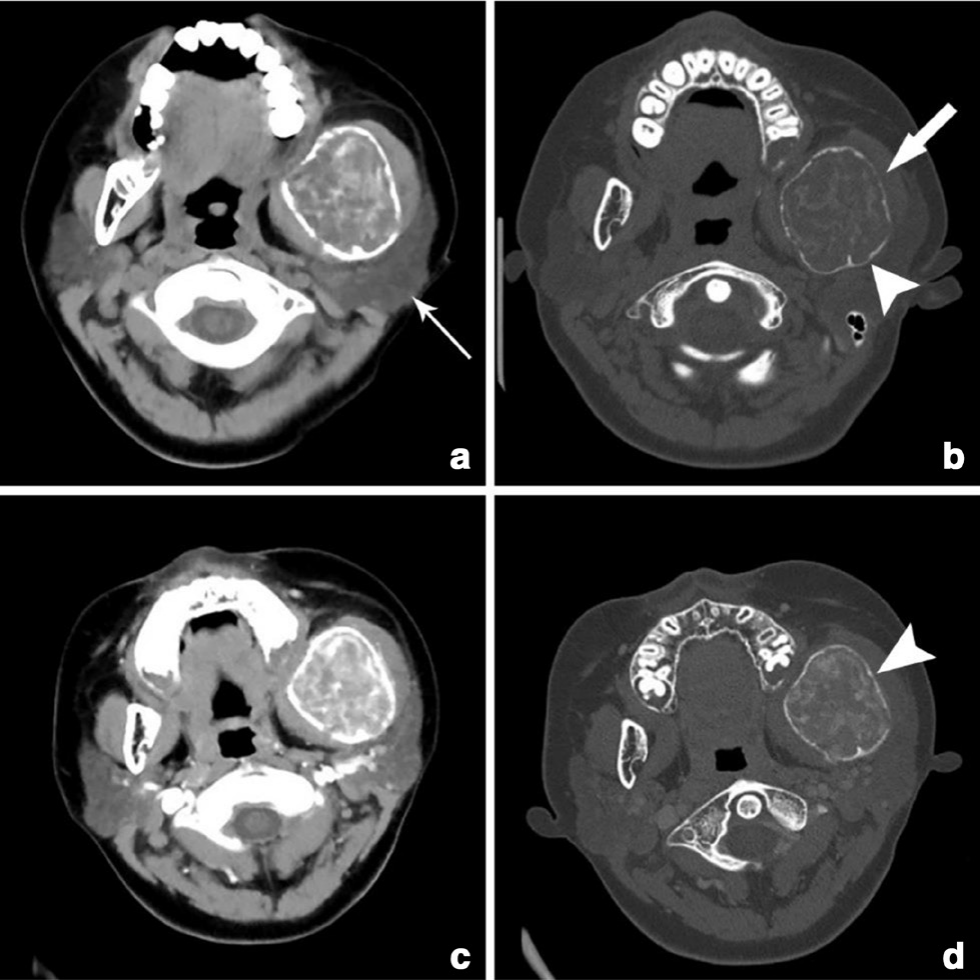
(a–d) Case 1: axial CT images without contrast (**a, **b) and with contrast (**c, **d) demonstrating expansile bony lesion of left mandible. The mass is well-circumscribed, radiolucent, cortical destruction and thinning (arrow head), granular bone pattern laterally (thick arrow). The adjacent soft tissue and masseter muscles are compressed and swollen (thin arrow).

All the three cases with lesion in the mandible showed relatively ill-defined, destructive lesion in the mandible causing mandibular ramus destruction along with swollen masseter muscle with low density shadows. Among them, 10-year-old female patient with history of trauma 3 months back and started swelling in face, since then along with difficulty in swallowing, had demonstrated an expansile bony lesion of the left mandible measuring 5 cm x 5 cm x 3 cm originating from the angle of the left mandible (Figure 1). On plain NECT scan, the lesion was well-circumscribed, radiolucent shadow with cortical bone destruction, cortical thinning and granular bony pattern laterally were present (Figure 1A and B). The expansile mass was compressing the adjacent soft tissue and masseter muscles. On CECT scan, the lesion was mildly and unevenly enhanced (Figure 1C and D).

**Figure 2.**
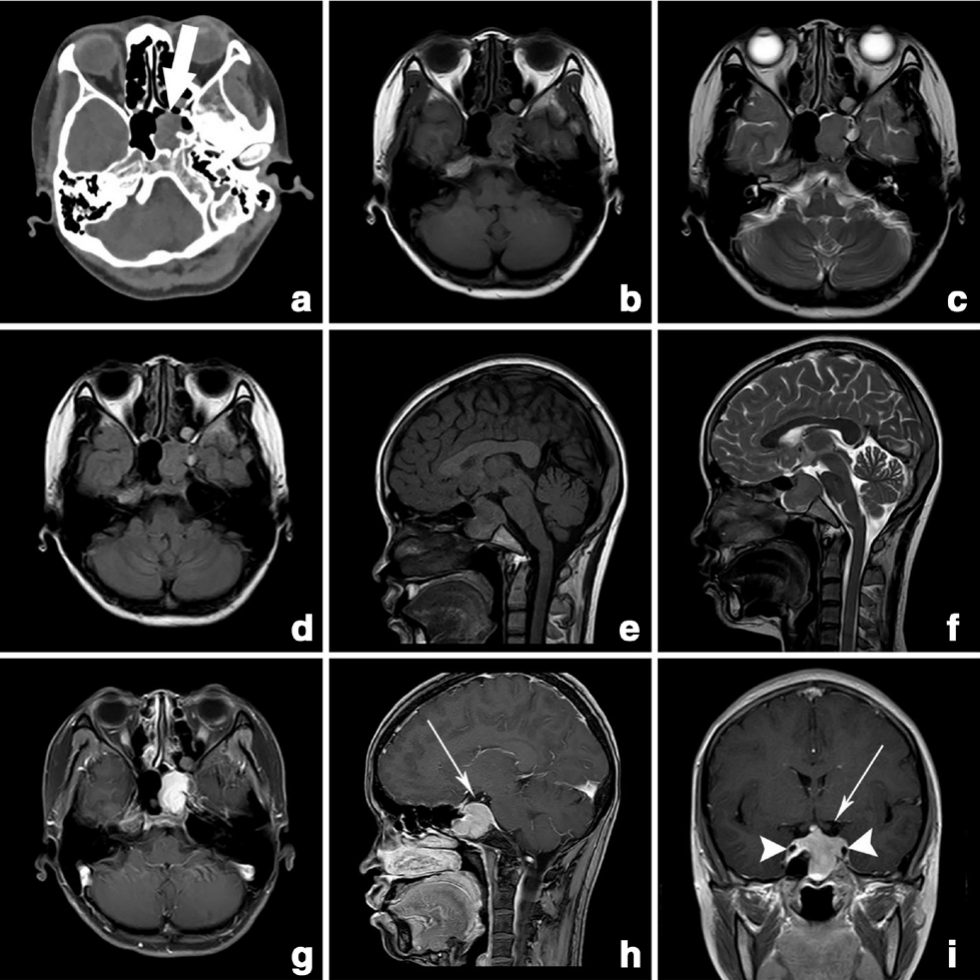
(a–i) Case 2: axial non-enhanced CT images (a) and MR images; axial *T*_1_WI (b), axial *T*_2_WI (c), axial FLAIR (d), sagittal *T*_1_WI (e), sagittal *T*_2_WI (f) and contrast enhanced *T*_1_WI axial (g), sagittal (h) and coronal (i) demonstrating expansile mass in the left sella turcica. The mass had low density with adjacent sphenoid bone destruction and cortical thinning (thick arrow) with displacement of optic chiasma (thin arrow) and bilateral internal carotid arteries (arrow heads). Contrast MR images demonstrated uniform enhancement in the lesion (**g–i**). FLAIR, fluid attenuated inversion recovery; *T*_1_WI, *T*_1_ weighted image; *T*_2_WI, *T*_2_weighted image.

Both the two cases with lesions in the sellar region showed low density space occupying soft tissue lesions. The expansile lesions had destructed the sellar bones and there was obvious thinning of cortex. The lesions had pushed optic chiasma upward but hadn’t compressed it in both cases. Among them, 25-year-old female patient with history of headache and vomiting for 1 month duration without any past history of trauma had demonstrated an expansile mass with uneven density in the sellar region originating from left sided sella turcica measuring 3 cm x 2.8 cm x 2 cm (Figure 2). The lesion caused peripheral sphenoid bone destruction and thinning of bony cortex (Figure 2a). MRI showed the lesion with low to iso-intensity signals in *T*_1_WI ((Figure 2b, e) and *T*_2_WI (Figure 2c, f), intermediate signals in *T*_2_ fluid attenuated inversion recovery (FLAIR) image (Figure 2d) and uniform enhancement after gadolinium contrast enhancement (Figure 2g, i). The mass had displaced optic chiasma and bilateral internal carotid artery upward and but hadn’t compressed them.

**Figure 3.**
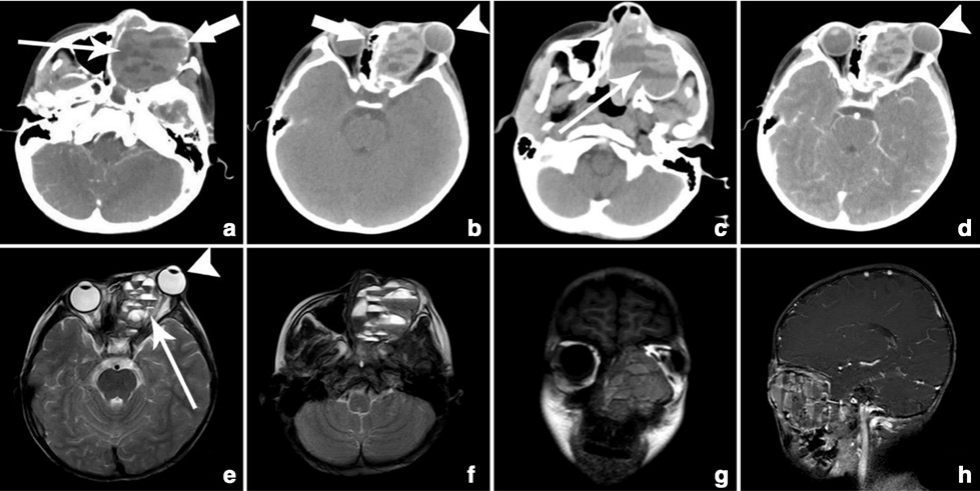
(a–h) Case 3: axial non-enhanced CT images (**a, **b) and contrast enhanced CT images (**c, **d), axial MR *T*_2_WI (**e, **f) and coronal MR *T*_1_WI (g) and contrast enhanced sagittal MR *T*_1_WI (h) demonstrating uneven density soft tissue mass arising from maxillary bone and expanded into the maxillary sinus, left orbital cavity, paranasal sinuses and within nasal cavity resulting in protrusion of left eyeball (arrow head), destruction of adjacent bony structures (thick arrow), multiple fluid levels (thin arrow) and deviated nasal septum towards the right side. *T*_1_WI, *T*_1_ weighted image; *T*_2_WI, *T*_2_weighted image

One case had lesion in the maxillary region which was located in the maxillary sinus extended growth into the paranasal sinuses, nasal cavity, orbital region and even caused protrusion of left eyeball. This was the case of 7-year-old male patient with a history of protrusion of left eye, blurring of vision, itching over orbital region and nasal blockage for 1 year of duration. The mass was 4.8 cm x 5.3 cm x 5.9 cm in size arising from left maxillary bone and expanded into the maxillary sinus, left orbital cavity, paranasal sinuses and within nasal cavity (Figure 3). The lesion showed soft tissue density in the CT with multiple fluid planes within the lesion. The lesion showed slight enhancement and had bone destruction around the maxillary sinus, nasal septum and inferior orbital wall (Figure 3A–3D). It resulted in protrusion of left eyeball outward, deviation of nasal septum, blockage of nasal cavity and paranasal sinuses. MRI showed multicystic lesion with multiple fluid–fluid levels within the lesion in *T*_2_WI (Figure 3E and F) and iso to slightly high signal in *T*_1_WI (Figure 3G) and unevenly enhancement present within the lesion with peripherally mild enhancement of cystic walls in contrast study (Figure 3H). The imaging features were similar to the features of aneurysmal bone cysts (ABC) but, histopathological investigation confirmed to be CGCG.

## Discussion

GCG are of two forms; peripheral giant cell granuloma (PGCG) and CGCG.^6–9^ These two different groups of pathological entities of GCG has similar histological features but pathogenesis of both are still not clear. PGCG are also reactive and exophytic lesion arising from extraosseous tissues (soft gum tissues). It is not a true neoplasm but thought to be as a result of chronic irritation to the area due to local irritation or trauma. Whereas, CGCG are intraosseous and non-proliferative lesions and non-neoplastic lesions. It is less common than PGCG and are commonly manifested in mandible and maxillary bones. Histologically, PGCG and CGCG both are similar. However, they differ in terms of aggressiveness as CGCG are more aggressive and higher recurrence tendency than PGCG.^6,8,9^

CGCG can display variable clinical presentation, including slow asymptomatic growth without recurrence to fast painful growth with perforation of cortical bone plate and ulceration to the mucosal surface. It can be present in patients from age of 2 years above. But most cases are seen between 20 and 40 years of age.^1,5^ Females are affected slightly more than males, the reason for this is thought to be because of hormonal factors despite the fact that lesions rarely express estrogen receptors.^6,10^

CGCG are more commonly located in the anterior portion of mandible and often crosses the midline. But the literatures have reported its occurrence in different locations like hard palate, orbital region, para nasal sinus, nasal cavity and septum, metacarpal bones and phalanges etc, which indicates that it can occur anywhere in the body.^6,11,12^

The etiology of CGCG is still not certain and has many theories for its pathogenesis.^12,13^ Previously, it was considered to be a hyperplastic reparative reaction to the intraosseous hemorrhage induced by trauma. However, a definite history of trauma may not be reliably elicited. Other theories on pathogenesis of CGCG including infectious and repair process, developmental disturbance, or even inflammatory causes had been proposed, but no single theory has been widely accepted.^12^ It has also been hypothesized as genetic etiology but lacks the convincing evidence to support the hypothesis.^5,6^

The most common clinical manifestations of CGCG include pain, swelling and palpable bone lesions and symptoms can vary according to the site of the lesion. In our study, apart from pain and swelling, patients had presented with headache, nausea and vomiting, visual disturbance, protrusion of eyes etc as per the location of the lesion.

Histological examination of CGCG suggests the lesions are composed of hypercellular fibrous stroma containing numerous multinucleated giant cells within the background of mononuclear stromal cells and spindle shaped fibroblasts along with areas of hemorrhages or foci of cystic degeneration and osteoid production.^5,7,11,12^ The histologic findings of CGCG, giant cell tumor (GCT) and brown tumor of hyperparathyroidism have virtually identical histologic features and closely resembles granulomas. Immunohistochemical studies have reported CGCG positive for cluster differentiation (CD) 68.^5,6^ In our study also, four cases of Immunohistochemistry were positive for CD 68.

Radiographically, CGCG appearance ranges from unilocular to multilocular radiolucent well-defined to ill-defined margins. The lesion bony defects size and nature varies according to the aggressiveness of the lesion. Moreover, the lesions may cause damage to adjacent structures like, displacement of teeth, tooth root resorption, cortical bone perforation.^6,8,12^ Chuong et al and Ficarra et al has classified CGCG into aggressive and non-aggressive types on the basis of six criteria like pain, growth rate, swelling, tooth root resorption, cortical perforation and recurrences.^14,15^ Aggressive lesions exhibit pain and rapid growth and usually more than 5 cm in size with the features of swelling and cortical bone perforation and teeth displacement and root resorption.^4,6,9,16^ And this type of lesion has high chances of recurrence. Whereas, non-aggressive lesions are low growing and have no or less symptoms and may be without associated features.

CT bone algorithm demonstrated that all the patients had the lesions showed lytic bone destruction and with remodeling of adjacent bone. CT scan revealed well-circumscribed expansile mass with the presence of subtle granular bone pattern at the periphery of the expanded bone with some internal septa.^16^ All lesions appeared to be multilocular and in one case, there was presence of multiple fluid levels within the lesions. The lesions showed heterogeneous soft tissue attenuating masses with mild enhancement on contrast study. MRI have some advantages over CT due to its high soft tissue differentiation and multiplanar depictions. MR images reveal a soft tissue area of low signal intensities on both *T*_1_- and *T*_2_ weighted images along with variable intensities within lesion if there is presence of fibrosis, osteoid, hemorrhage or hemosiderin deposits. Post-gadolinium MR images of the lesion can show marked enhancement but the degree of enhancement can vary.^12^ These CT and MR imaging features are not specific but the features can be suggestive of the disease. These imaging features may be indistinguishable from GCT, ABCs and brown tumor of hyperparathyroidism.

## Differential diagnosis

The differential diagnosis of CGCG includes aneurysmal bone cyst, benign chondroblastoma, brown tumor of hyperparathyroidism, cherubism, fibrous dysplasia, non-osteogenic fibroma, osteosarcoma and true GCT.^5,7,12^

Fibrous dysplasia and other odontogenic tumors and non-odontogenic tumors can be easily ruling out on the basis of their clinical and radiological features and histopathology.^11^

Brown cell tumor of hyperthyroidism usually occurs later in the life and is characterized by multiple lesions. Parathyroid hormone, serum and urinary levels of calcium, phosphate and bone or serum alkaline phosphatase are used in the diagnosis of brown cell tumors.^7,8,11,12^

ABCs are non-neoplastic lesions in the bone containing giant cells. Radiographs show multiple cystic cavities filled with blood within thin walls. MRI reveals a heterogeneous high signal intensity lesion but histologically, they are characterized by thin-walled blood-filled sinuses lined by fibroblasts and giant cells.^6,8,17^

Chondroblastoma of the temporal bone is a locally aggressive tumor and histologically, it is characterized by presence of hemosiderin pigment, chondroid differentiation, scattered giant cells and calcification and can appear as high-density mass on CT scan.^8,12^

While differentiation between GCT and CGCG is often difficult and confusing. GCTs are benign and locally aggressive true neoplasm. They have an incidence of 3–7%. And among them only 2% of GCTs occur in skull.^5^ It occurs in third to fourth decade of life while symptoms vary according to the site of the lesion. These tumors can go into malignant transformation..^5,8,12,18^ CGCG and GCT origin are different. CGCG origins from periosteal connective tissue while GCT originates from bone marrow connective tissue. But both the lesions are composed of multinucleated giant cells and small oval or spindle shaped fibroblasts.^6,17^ CGCG can be differentiated from GCT on the basis of histopathological features as; CGCG have relatively fewer multinucleated giant cells than GCT with increased incidence of osteoid, fresh hemorrhages and hemosiderin deposits. In contrast, the giant cells are more evenly distributed in GCT. Other features of CGGC include increased fibrosis, increased spindle-shaped fibroblasts and absence of necrosis.^5,11,12,17^ However, considerable overlap of characteristics can occur between these two lesions.

## Treatment

Surgical management is the most common treatment modality employed. It can be done by two different procedures: curettage ± adjunctive treatment (*e.g.* cryotherapy, osteotomy etc) and resection. All patients in this study had underwent surgical resection and no recurrence cases were observed till now on regular follow-up. Literatures have reported the higher recurrence rate of curettage (33–75%) so, total surgical resection is considered to be the best one for CGCG with lesser recurrence rate (10–20%).^7,19,20^ Post-surgical morbidity may be high often leading to severe aesthetic and functional problems. Unfortunately, medical management are found to be ineffective in treating CGCG. Different medical approaches including α interferon, calcitonin and intralesional corticosteroid injections have been evolved over last years to avoid mutilating surgery as well as to reduce recurrence after surgical management. Denosumab therapy has been successfully used to treat patients with GCTs of bone and also has shown successful results in treating CGCG.^21^ However, there is very limited numbers of case studies in this matter, the long-term use of denosumab has shown some promising results. Intranasal calcitonin spray as maintenance therapy has also shown significant result in preventing recurrence after surgical curettage.^20,21^ Combination of denosumab and intranasal calcitonin may also increase treatment response and reducing in recurrence of CGCG rate, but the sufficient studies are still lacking. Radiotherapy can also be used for CGCG cases where surgery is difficult to perform, but literatures have reported the higher chances of malignant transformation post-radiotherapy.^8,9^ For the close monitoring of recurrence of CGCG after surgical treatment, CT and MRI should be done on follow-up regularly.

## Conclusion

CGCG are non-neoplastic and non-proliferative intraosseous lesions with unclear pathogenesis. It can occur in any part of body and shows symptoms as per the location of the lesion. It can be diagnosed histologically as well as radiographically. But, the disease features are similar to other common diseases like GCT, Brown tumor of hyperthyroidism etc. Radiographically, lesion with unevenly dense expansion along with bone destruction and cortical thinning with low to iso-intensity in *T*_1_WI and *T*_2_WI and mildly enhanced periphery, CGCG should also be considered.
